# Impact of the reduction in TSH cutoff level to 6 mIU/L in neonatal screening for congenital hypothyroidism in Santa Catarina: final results

**DOI:** 10.20945/2359-3997000000299

**Published:** 2020-10-21

**Authors:** Marilza Leal Nascimento, Andre Leal Nascimento, Patricia Dornbusch, Masanao Ohira, Genoir Simoni, Edson Cechinel, Rose Marie Mueller Linhares, Juliana van De Sande Lee, Paulo Cesar Alves Silva

**Affiliations:** 1 Hospital Infantil Joana de Gusmão Departamento de Endocrinologia Pediátrica Florianópolis SC Brasil Hospital Infantil Joana de Gusmão, Departamento de Endocrinologia Pediátrica, Florianópolis, SC, Brasil; 2 Universidade Federal de Santa Catarina Florianópolis SC Brasil Universidade Federal de Santa Catarina (UFSC), Florianópolis, SC, Brasil

**Keywords:** Congenital hypothyroidism, neonatal screening, TSH

## Abstract

**Oubjective:**

To assess the implications of changing the cutoff level of TSH from 10 to 6 mIU/L.

**Subjects and methods:**

The study population was constituted by 74.123 children screened for congenital hypothyroidism by the National Screening Program in Santa Catarina, from March 2011 to February 2012. The cutoff of TSH was 6 mIU/L. If TSH between 6-10 mIU/L, the newborn was recalled for a second TSH measurement on filter paper. If TSH > 6 mIU/L in the second sample, the child was sent for medical evaluation. In children with normal topic thyroid, levothyroxine was suspended for 1 month at the age of 3 years for identification of the etiology and evaluation of the need to continue treatment.

**Results:**

Among the children screened, 435 were recalled for presenting TSH between 6 and 10 mIU/L in the first sample, 28 remained TSH > 6 mIU/L in the second sample. Among these, 11 had a final diagnosis of dyshormonogenesis, two of ectopic thyroid, two of thyroid hypoplasia and one of transient hypothyroidism. Ten children presented normal TSH levels on the first medical evaluation and two lost follow-up.

**Conclusion:**

A decrease in the TSH cutoff level from 10 to 6 mIU/L in a neonatal screening program for congenital hypothyroidism reduced the number of false-negative results, increasing the sensitivity of the test, but increased the number of false-positive results and recalls. Since a TSH cutoff level of 6 mIU/L detects thyroid function abnormalities requiring treatment, the adoption of this cutoff level is justified.

## INTRODUCTION

More than a century ago, the treatment of congenital hypothyroidism progressed considerably with the use of thyroid extracts. At that time, the diagnosis of severe cretinism was based only on clinical observation, and the success of treatment was reflected on a dramatic improvement in linear growth. The absence of cognitive improvement at that time suggested a critical role for thyroid hormones in neuronal maturation during a narrow window of time at the beginning of life ( 1 ). Indeed, the deficiency of thyroid hormones in congenital hypothyroidism affects the newborn's growth and maturation and impacts the development of the central nervous system ( 2 , 3 ).

Congenital hypothyroidism is one of the most common preventable causes of mental retardation ( 1 - 4 ). Early diagnosis of this condition based on clinical manifestations alone is challenging due to the absence or lack of specificity of signs and symptoms of the disease in the neonatal period. Indeed, less than 5% of the cases of congenital hypothyroidism are clinically recognized at birth ( 5 ).

Treatment of congenital hypothyroidism should ideally initiate before 14 days of life. Timely diagnosis and treatment of children affected by this condition yields substantial benefits for both family and community and prevents social, emotional, and financial burden associated with mental disability ( 1 - 6 ). Neonatal screening programs have been developed for early detection of diseases leading to neurological sequelae, including congenital hypothyroidism ( 2 , 4 , 6 - 8 ).

Congenital hypothyroidism affects 1:3.000 to 1:4.000 newborns worldwide ( 2 , 4 - 8 ). It affects girls and boys at a ratio of 2:1 and has an increased prevalence in children with Down's syndrome ( 2 ). In Santa Catarina, congenital hypothyroidism affects 1 in every 3.035 newborns and has a higher prevalence in females compared with males (2:1) ( 9 ).

Over the past decade, several neonatal screening programs have reported an increasing prevalence of congenital hypothyroidism from 1:2.000 to 1:4.000 or higher. Several factors seem to contribute to this effect, including the reduction in TSH cutoff levels in neonatal screening programs ( 4 , 7 , 8 ).

Congenital hypothyroidism may be permanent or transient. Children who screen positively for congenital hypothyroidism at birth and start treatment with levothyroxine while having no established etiology for the disease are recommended to have levothyroxine suspended and be reevaluated at the age of 3 years ( 3 ).

The neonatal screening program of the state of Santa Catarina (PTN-SES/SC), Brazil, was implemented in July 1992 and includes screening for congenital hypothyroidism. The program initially adopted a TSH cutoff level of 10 mIU/L. Later, a study carried out between April 2005 and March 2007 in the United Kingdom ( 10 ) reduced the TSH cutoff level from 10 mIU/L to 6 mIU/L. In the study, 67 children born at term presented TSH levels between 6.1 mIU/L and 10 mIU/L in the initial test, and four persisted with TSH above 6 mIU/L in a second evaluation, two of whom were diagnosed with congenital hypothyroidism.

Another study conducted between May 2005 and October 2007 at the Ribeirão Preto Medical School (FMRP) in São Paulo reduced the TSH cutoff value to 5 mIU/L ( 11 ). Of 76,800 screened newborns, the decreased cutoff value identified seven children with congenital hypothyroidism who would otherwise be missed if the TSH level for screening was set at 10 mIU/L ( 11 ).

Neonatal screening programs must meet certain characteristics like a low rate of recalls or false-positive results and a reduced number of losses or false-negative results ( 1 , 2 , 4 ). A TSH cutoff value of 10 mIU/L increases the number of losses or false-negative results, while a value of 6 mIU/L increases the number of recalls or false-positive results but reduces the number of false-negative results.

Considering these observations, the objective of this study was to assess the impact of a reduction in TSH cutoff level from 10 mIU/L to 6 mIU/L in the PTN-SES/SC. Results of this study should provide data for improvement of neonatal screening programs.

## SUBJECTS AND METHODS

This retrospective cohort study was approved by the Research Ethics Committee of Plataforma Brasil (CAAE 53097416.4.0000.5361).

The study population comprised all 74,123 children screened for congenital hypothyroidism by the PTN-SES/SC between March 2011 and February 2012.

For the initial screening, TSH was measured from blood collected by heel puncture on specialized linear filter paper (100% cotton, thickness 2.24 mm, basis weight 105-110 g/m^2^; Fitec^®^, Fitec Filters, Jandira, SP). The blood was collected by the nursing staff in hospitals and health care centers in all municipalities across Santa Catarina. The samples were mailed to or delivered in person at the Sector of Neonatal Analyses at the Central Laboratory (LACEN), in Florianópolis. Samples were ideally collected between the third and fifth days of life.

TSH was quantified by time-resolved immunofluo-rometric assay in an automated immunoassay system (1235 AutoDELFIA, PerkinElmer, Inc., Waltham, MA, USA) using the AltoDELFIA Neonatal hTSH kit.

The cutoff value adopted for TSH measurement was 6 mIU/L. Newborns with a value between 6 and 10 mIU/L were recalled for a second TSH measurement on filter paper. Children with a second measurement < 6 mIU/L were considered normal and were dismissed, while those with results ≥ 6 mIU/L were referred for medical evaluation.

All children referred for medical evaluation were seen at the pediatric endocrinology outpatient clinic of *Hospital Infantil Joana de Gusmão* (HIJG) in Florianópolis (Santa Catarina). During the first appointment, the child was evaluated with a detailed history taking and examined by one of the six physicians of the team. On physical examination, the presence of the following manifestations was investigated: hoarse cry, macroglossia, umbilical hernia, prolonged jaundice (> 7 days), constipation, drowsiness, large anterior fontanel, globose abdomen, hypotonia, goiter, hypoactivity, cold skin, paleness, livedo reticularis, infiltrated skin, enlarged nasal base, ocular hypertelorism, and breastfeeding problems. After history taking and physical examination, complementary tests were performed, including serum measurement of TSH (for confirmation of congenital hypothyroidism), free T4 (fT4), and thyroglobulin (TG), in addition to knee X-ray and thyroid ultrasonography. All result, with the exception of TG, were obtained on the same day of the appointment. We considered as normal those values of TSH < 6 mIU/L, fT4 between 0.73 and 2.01 ng/dL, and TG between 2.0 and 35.0 ng/dL. If the results of the tests confirmed the diagnosis of congenital hypothyroidism (TSH > 10 mIU/L), treatment with levothyroxine 10-15 μg/kg/day was initiated. Children with TSH of 6-10 mIU/L and normal ultrasound were monitored every 15 days without treatment and were discharged if the TSH levels normalized, or were started on treatment with levothyroxine if the levels increased.

Knee X-ray for evaluation of bone age was obtained with the device THA BI 150/30/51-100 (Siemens, Erlangen, Germany) in the radiology department at HIJG. Bone age was considered delayed when the distal epiphyseal nucleus of the femur was absent in a child born full-term or with a corrected gestational age above 37 weeks.

Thyroid ultrasound was performed at the radiology department of HIJG with the ultrasound equipment EPIQ 5G and a linear 7.5- to 10-MHz transducer (Philips Medical Systems, Bothell, WA, USA). According to the ultrasonographic evaluation, the thyroid was classified as (A) absent, (B) ectopic, (C) normal topic, (D) increased in volume, or (E) reduced in volume.

The etiology of the congenital hypothyroidism was determined based on ultrasound assessment and serum fT4 and TG levels, according to the protocol identified in Table 1 .

**Table 1:** Etiological investigation based on ultrasound evaluation and measurement of serum free T4 and thyroglobulin in newborns with elevated TSH levels

Low thyroglobulin	Measurable thyroglobulin	Measurable thyroglobulin
Low free T4	Low or normal free T4	Low or normal free T4
Thyroid undetectable on ultrasound	Thyroid ectopic or undetectable	Thyroid topic and visible
↓	↓	↓
Agenesis	Ectopy	Normal gland (dyshormonogenesis or transitional congenital hypothyroidism)
		Hypoplasia
		Hemithyroid

Adapted from Grüters ( 29 ).

In patients with a normal topic thyroid, treatment with levothyroxine was interrupted for 1 month at the age of 3 years, when serum TSH levels were measured again to evaluate if the hypothyroidism was transient or permanent (dyshormonogenesis). Values of TSH above 9.9 mIU/L on this evaluation confirmed the diagnosis of dyshormonogenesis. Levels of TSH consistently below 5 mIU/L on three serial evaluations with 1-month intervals between each during treatment suspension confirmed transient hypothyroidism. Patients who presented with TSH levels between 5 and 9.9 mIU/L were reassessed with monthly TSH measurements; an increase in TSH levels established the occurrence of dyshormonogenesis. Once an etiological diagnosis of dyshormonogenesis was established, treatment with levothyroxine was restarted.

### Statistical analysis

The data were stored and analyzed using the software Microsoft Office Excel 2007^®^ (Microsoft, Seattle, WA, USA). The results are described in absolute frequency and percentage, mean, and standard deviation values. Fisher's exact test was used to verify the association between nominal qualitative variables. Quantitative variables with and without a normal distribution were analyzed with Student's *t* and Mann-Whitney U test, respectively. P values < 0.05 were considered significant.

## RESULTS

Of the 84,287 live births in the state of Santa Catarina between March 2011 and February 2012 ( 12 ), 74,123 were screened by the PTN-SES/SC, totaling a program coverage of 87.94%.

Among the screened children, 435 (0.6%) had TSH levels between 6 and 10 mIU/L in the first measurement and were recalled for a second TSH measurement on filter paper, of whom 28 (6.4% of the 435 recalled children) maintained TSH > 6 mIU/L in the second measurement on filter paper and were referred for medical evaluation. Of these 28 children, the serum TSH levels normalized in 10, while 18 were diagnosed with congenital hypothyroidism and treated with levothyroxine. In this group, 24 children were recalled for a new TSH measurement for each confirmed case of congenital hypothyroidism.

Of the 18 children with confirmed hypothyroidism, 4 (22.2%) had thyroid dysgenesis (two had ectopic thyroid and two had thyroid hypoplasia), and 14 (77.8%) had a normal topic thyroid on ultrasonography. The distinction between transient hypothyroidism and dyshormonogenesis in these children was not possible during the initial care, and their etiologic diagnosis was determined at the age of 3 years ( 13 ).

Of the 14 patients whose definitive diagnosis was established at the age of 3 years, one was lost to follow-up, and one died at the age of 2 years due to severe congenital heart disease. In the remaining cases (12 patients) with normal topic thyroid, treatment with levothyroxine was discontinued for 1 month at the age of 3 years, and a final diagnosis of dyshormonogenesis was established in 11 cases (68.75%) and transient hypothyroidism in one case (6.25%).

Among the 18 children with congenital hypo-thyroidism, the distribution of the cases was 1 girl for each 1.5 boys, the mean age at the first measurement was 7.16 ± 3.61 days (95% CI), the mean TSH values in the first and second measurement were 7.26 ± 0.45 mIU/L and 8.21 ± 0.57 mIU/L, respectively, and the mean age at the first appointment was 26.77 ± 4.59 days. The group in which the TSH normalized had a ratio of girls to boys of 1:1.5, mean age at the first TSH measurement of 6.80 ± 1.51 days, mean TSH value in the first and second measurements of 7.60 ± 0.71 mIU/L and 7.17 ± 0.86 mIU/L, respectively, and a mean age at the first appointment of 27.77 ± 5.95 days.

During the first appointment, the main sign of hypothyroidism was umbilical hernia, which was present in nine patients (32.13%). Large anterior fontanel was observed in five patients (17.85%), while three patients (10.71%) presented prolonged jaundice and six were asymptomatic (21.42%). All three (10.71%) patients in whom TSH levels normalized had umbilical hernias.

Among 17 patients with congenital hypothyroidism evaluated with knee X-ray, bone age was normal in 11 and delayed in the remaining six.

The mean laboratory TSH measurement in the first appointment of the 18 children with confirmed hypothyroidism was 21.27 ± 7.31 mIU/L (range 6.5–60 mIU/L). Among those children in whom TSH normalized, the mean TSH value was 5.71 ± 1.48 mIU/L (range 1.97–8.49 mIU/L). Serum TSH levels in children with confirmed hypothyroidism were significantly higher than those in children with TSH normalization ( Table 2 ). The mean fT4 and TG levels among children with confirmed congenital hypothyroidism were 1.79 ± 1.09 ng/dL and 147.60 ± 80.16 ng/dL, respectively.

**Table 2:** Measurement of TSH levels on neonatal screening and serum TSH, free T4, and thyroglobulin levels in newborns with TSH normalization and confirmed diagnosis of congenital hypothyroidism (CH)

Variables	TSH normalization * ( 10 )	Confirmed CH * ( 18 )	P value
TSH on neonatal screening (filter paper) (mIU/L)	7.60 (6.89 to 8.31)	7.26 (6.81 to 7.71)	0.08 ‡
Serum TSH (mIU/L)	5.71 (4.23 to 7.19)	21.27(13.96 to 28.58)	0.000084 †
Free T4 (ng/dL)	1.24	1.79 (0.7 to 2.88)	0.79 ‡
Thyroglobulin (ng/dL)	85.08	147.60 (67.44 to 227.76)	0.49 ‡

* Values presented as mean (95% confidence interval).

† Student's *t* test.

‡ Mann-Whitney U test.

The mean age at treatment initiation was 31.66 ± 0.52 days (95% CI), and the maximum and minimum ages were 59 days and 15 days, respectively.

In patients with a final diagnosis of dyshormo-nogenesis, TSH measurements performed at the age of 3 years (after suspension of levothyroxine for 1 month) showed a mean value of 18.73 ± 4.82 mIU/L (range 10.32–37.75 mIU/L). The only patient with a final diagnosis of transient hypothyroidism had a TSH of 0.92 mIU/L 1 month after levothyroxine was suspended.

There were no significant differences in regard to sex, family history of thyroid disease, delivery route, or bone age between the group with TSH normalization in the first medical assessment and the group with confirmed congenital hypothyroidism (p value for all > 0.05).

## DISCUSSION

Neonatal screening programs for congenital hypothyroidism have been created to circumvent the challenges of establishing an early diagnosis of the disease based on clinical parameters alone and to initiate timely treatment to prevent mental retardation ( 2 ). The main objectives of neonatal screening programs are to cover 100% of the births, establish an early diagnosis, and deliver timely treatment to prevent sequelae ( 2 , 4 ).

The coverage of the PTN-SES/SC during the period of the study was 87.94%, which was superior to the coverage of the same program in 2009 (84.67%) ( 9 ) and in other Brazilian states like Rio de Janeiro (80.4%) ( 14 ), Bahia (71.52%) ( 15 ), Mato Grosso (67.6%) ( 16 ), and Sergipe (76.8%) ( 17 ). The program is unable to achieve 100% coverage since some screening tests are carried out in private laboratories, and the amount of these tests or the number of positive results is not notified to the state. This problem also occurs in other states since no legislation requires notification by these private laboratories ( 6 , 15 , 17 ).

Although congenital hypothyroidism is included in neonatal screening programs worldwide, there is a lack of consensus about the ideal TSH cutoff value for screening. This results in large variations in TSH cutoff values in different countries and, sometimes, even within the same country. Despite this lack of consensus, the cutoff values used in the various existing neonatal screening programs began to decrease slowly over the years ( 2 , 4 ).

Cutoff levels between 5 and 10 mIU/L have been adopted by some programs, including those in the United Kingdom ( 10 ), Slovenia, Norway ( 18 ), Italy ( 19 ), and Iran ( 20 ), with the aim of decreasing false-negative rates, but have increased the number of false-positive results and recalls ( 4 , 8 , 21 ).

A study conducted in the United Kingdom ( 10 ) reduced to 6 mIU/L the cutoff value of TSH for the screening of congenital hypothyroidism. Children with TSH levels between 6 and 20 mIU/L underwent a second TSH evaluation and when the levels remained above 6 mIU/L, the child was referred to clinical and laboratory evaluation. Those in whom the first TSH level was greater than 20 mIU/L were referred directly to medical evaluation. The study found that of 67 children with TSH levels between 6.1 and 10 mIU/L in the initial test, four remained with levels above 6 mIU/L in the second evaluation (including one with TSH > 10 mIU/L). Two were diagnosed with congenital hypothyroidism and started on levothyroxine. A similar study conducted at HCFMRP in São Paulo between May 2005 and October 2007 ( 11 ) also decreased the TSH cutoff value to 5 mIU/L. Children with levels < 5 mIU/L were considered normal, while those with levels between 5 and 10 mIU/L were considered borderline and underwent a second TSH measurement on filter paper. When TSH levels remained > 5 mIU/L in this second evaluation, the child was evaluated with serum TSH measurement. If ≤ 4 mIU/L, the child was considered normal and if > 4 mIU/L, the child was examined and followed up. Of the 76,800 children assessed, seven were diagnosed with hypothyroidism and received treatment, which would not have happened if the cutoff level was set at 10 mIU/L.

Congenital hypothyroidism due to synthesis defects (dyshormonogenesis) is an autosomal recessive disorder with a similar incidence in both sexes. In contrast, defects in thyroid development (dysgenesis) are more frequent among females and account for approximately 80% of the cases of congenital hypothyroidism with a TSH cutoff level > 10 mIU/L ( 21 , 22 ). In the present study, 68.75% of the patients had a diagnosis of dyshormonogenesis established at the age of 3 years, and males were more affected than females (1.5:1).

At least one clinical sign of congenital hypothyroidism was present on physical examination in 53.3% of the newborns evaluated in our study. The most frequent manifestations were umbilical hernia, large anterior fontanel, and jaundice prolonged for more than 7 days. Umbilical hernia (51%) and large anterior fontanel (50.3%) were also predominant among children with congenital hypothyroidism in a study conducted in Minas Gerais ( 23 ). In our study, 21.42% of the newborns were asymptomatic, which confirms the challenge in establishing a timely diagnosis based on physical examination alone and the importance of neonatal screening and complementary tests.

The TSH values among confirmed cases of congenital hypothyroidism ranged from 6.5 mIU/L to 60 mIU/L (mean 21.27 mIU/L). With the previous cutoff value (10 mIU/L) adopted by the program, the diagnosis of congenital hypothyroidism in these children would have been missed in the neonatal period and probably only be established later on when physical, psychological, and motor manifestations emerged, and neurological sequelae were already irreversible.

Ultrasound is a fundamental tool in defining the etiology of the disease in congenital hypothyroidism, as shown in Table 1 . Together with measurement of serum TG, ultrasound may help distinguish between thyroid agenesis and ectopy ( 24 ). Measurable levels of fT4 and TG in the absence of thyroid tissue in the normal location of the gland on ultrasound suggests the occurrence of ectopic thyroid ( 24 , 25 ). A study has shown that serum TG (normal range 20 to 80 ng/mL) was present in low concentrations in children with thyroid agenesis (mean 12 ng/mL, range 2 to 54 ng/mL), while intermediate concentrations occurred in children with ectopic thyroid tissue (mean 92 ng/mL, range 11 to 231 ng/mL) and high concentrations in those with goiter (mean 226 ng/mL, range 3 to 425 ng/mL) ( 25 ). In this study, the mean TG level in children with congenital hypothyroidism referred to medical evaluation was 147.60 ± 80.16 ng/dL. In both patients diagnosed with ectopic thyroid, TG was measurable, and the gland was not visible on ultrasound.

Delayed bone age was observed in 54.54% of the children with congenital hypothyroidism at the first medical evaluation in the present study. Delayed bone age reflects a compromised thyroid function at birth, especially in severe cases of hypothyroidism, and relates to changes in neurological, psychological, and motor development during the first year of life, irrespective of other variables related to treatment ( 26 ).

Treatment of congenital hypothyroidism should be initiated early, ideally before 14 days of life, to prevent central neurological sequelae, mainly decreased intelligence (reduced intelligence quotient [IQ]) ( 27 , 28 ). In the present study, treatment was not initiated timely. Newborns with TSH ≥ 10 mIU/L in the first appointment were immediately started on treatment with levothyroxine 10-15 μg/kg. The remaining newborns diagnosed with congenital hypothyroidism were only started on treatment when subsequent TSH measurements were ≥ 10 mIU/L.

The adoption of a TSH cutoff level of 6 mIU/L in the present study resulted in an increased number of recalls and false-positive results compared with the cutoff level of 10 mIU/L previously adopted by the program. With a TSH cutoff level of 10 mIU/L, 8 children were recalled for a new blood sample collection for each child diagnosed with CH ( 9 ). However, four patients were diagnosed with thyroid dysgenesis, two of whom with ectopic thyroid and two with thyroid hypoplasia. In addition to these, 14 (two were lost to follow up) had a normal topic thyroid on ultrasonography and an initial differentiation between transient or permanent (dyshormonogenesis) hypothyroidism ( 13 ) was unfeasible. The etiology of the disease in these children was established at the age of 3 years after discontinuation of levothyroxine for 1 month, and the final diagnosis was dyshormonogenesis in 11 cases and transient hypothyroidism in one case.

With the exception of one patient with transient hypothyroidism, the TSH level after suspension of levothyroxine was above 10 mIU/L in all others, with a mean of 18.73 mIU/L (range 10.32–37.75 mIU/L), confirming the diagnosis of congenital hypothyroidism due to dyshormonogenesis and a need to maintain treatment with levothyroxine.

With the TSH cutoff value of 10 mIU/L previously adopted by the neonatal screening program in Santa Catarina, 4 children were recalled for a second TSH measurement on filter paper for each child diagnosed with congenital hypothyroidism ( 29 ). Using a cutoff value of 6 mIU/L, 24 children with TSH levels between 6 and 10 mIU/L were recalled for each child diagnosed with congenital hypothyroidism, increasing the cost of the program and the anxiety of the parents. A timely diagnosis of the disease yields substantial savings to the health care system. An Australian study conducted in 2005 estimated that AUD $1,590,701 were saved for each case of congenital hypothyroidism diagnosed early ( 30 ).

In 2009, the ratio of births affected by congenital hypothyroidism to normal ones in Santa Catarina was 1:3,035 ( 9 ). This ratio increased to 1:1,560 with a reduction of the TSH cutoff level to 6 mIU/L, a finding that is aligned with the increased prevalence in other screening programs that reduced the cutoff levels for TSH ( 4 , 10 , 11 , 19 , 21 ). An important fact is that a large part of the cases of congenital hypothyroidism responsible for this increased prevalence had a topic thyroid gland and milder thyroid dysfunction ( 7 , 8 , 31 ), as observed in the present study.

In conclusion, a reduction in the TSH cutoff level to 6 mIU/L in our neonatal screening program decreased the number of false-negative results and increased the sensitivity of detection of congenital hypothyroidism. However, it also increased the number of false-positive results and recalls, resulting in higher costs to the state and the families. Despite that, the adoption of 6 mIU/L as the cutoff level for TSH is justifiable as it allows early detection and management of a condition with dramatic consequences when left untreated.
